# Surgical management of a solitary metastatic ovarian adenocarcinoma with colonic origin presenting as gigantic bilateral ovarian masses

**DOI:** 10.1002/ccr3.6336

**Published:** 2022-09-12

**Authors:** Seyed Mohsen Ahmadi Tafti, Behnam Behboudi, Fatemeh Nili, Alireza Hadizadeh

**Affiliations:** ^1^ Division of Colorectal Surgery, Department of Surgery Tehran University of Medical Sciences Tehran Iran; ^2^ Colorectal Surgery Research Center, Imam Hospital Complex Tehran University of Medical Sciences Tehran Iran; ^3^ Department of Pathology, Imam Khomeini Hospital Complex Tehran University of Medical Sciences Tehran Iran; ^4^ Research Center for Advanced Technologies in Cardiovascular Medicine, Cardiovascular Diseases Research Center Institute Tehran University of Medical Sciences Tehran Iran; ^5^ School of Medicine Tehran University of Medical Sciences Tehran Iran

**Keywords:** colorectal cancer, gynecological malignancy, Krukenberg tumors, metastasis, ovarian cancer

## Abstract

Metastatic ovarian tumors with a gastrointestinal origin have always been a challenge in surgery; in many cases, the primary tumor is diagnosed after the metastasis. This case was presented with bilateral abdominal masses, which were adenocarcinomas originating from gastrointestinal tract. Following colonoscopy and finding a mass, total colectomy was performed.

## Abstract 2

The key clinical message, in this case, is that large, bilateral masses in the absence of ascites are suggestive for an extragonadal origin for which one of the primary sources is GI, so endoscopy and colonoscopy should be carried out for these patients (for gastric and colonic cancers). Resecting the primary tumor with metastasis is controversial, and not currently enough data are available. Our assumption is that we have to rule out the secondary sites of metastasis and peritoneal seeding, for which palliative chemotherapy and ostomy diversion for obstructive cases is the only option. In cases with no secondary site of metastasis surgery is considered an option, but the risk of metachronous metastasis is high, and the prognosis is poor.

## 1 INTRODUCTION

Ovarian cancer is the leading gynecological malignancy‐related death.^1^, ^2^ Ovarian cancers consist of at least five histopathological categories, and each type has its characteristic identifiable risk factors and origins.^3^, ^4^, ^5^ While most of these cancers are primary in origin, some types, such as Krukenberg tumors, are secondary metastasis from the GI tract.^1^, ^6^ These cases are often accompanied by peritoneal or liver metastasis. Still, rare cases of ovarian masses without systemic metastasis are also reported, and surgery is considered an option for these cases. These cases are considered “solitary ovarian metastatic tumors.” The appropriate and exact approach has not yet been defined in guidelines for these patients since these cases have a poor prognosis, and systemic metastasis is present in many of these patients.

Our case is a 26‐year‐old woman who was first presented with abdominal distention and palpable mass, which turned out to be ovarian adenocarcinoma originating from the colon after surgical intervention.

## 2 CASE PRESENTATION

**FIGURE 1:**
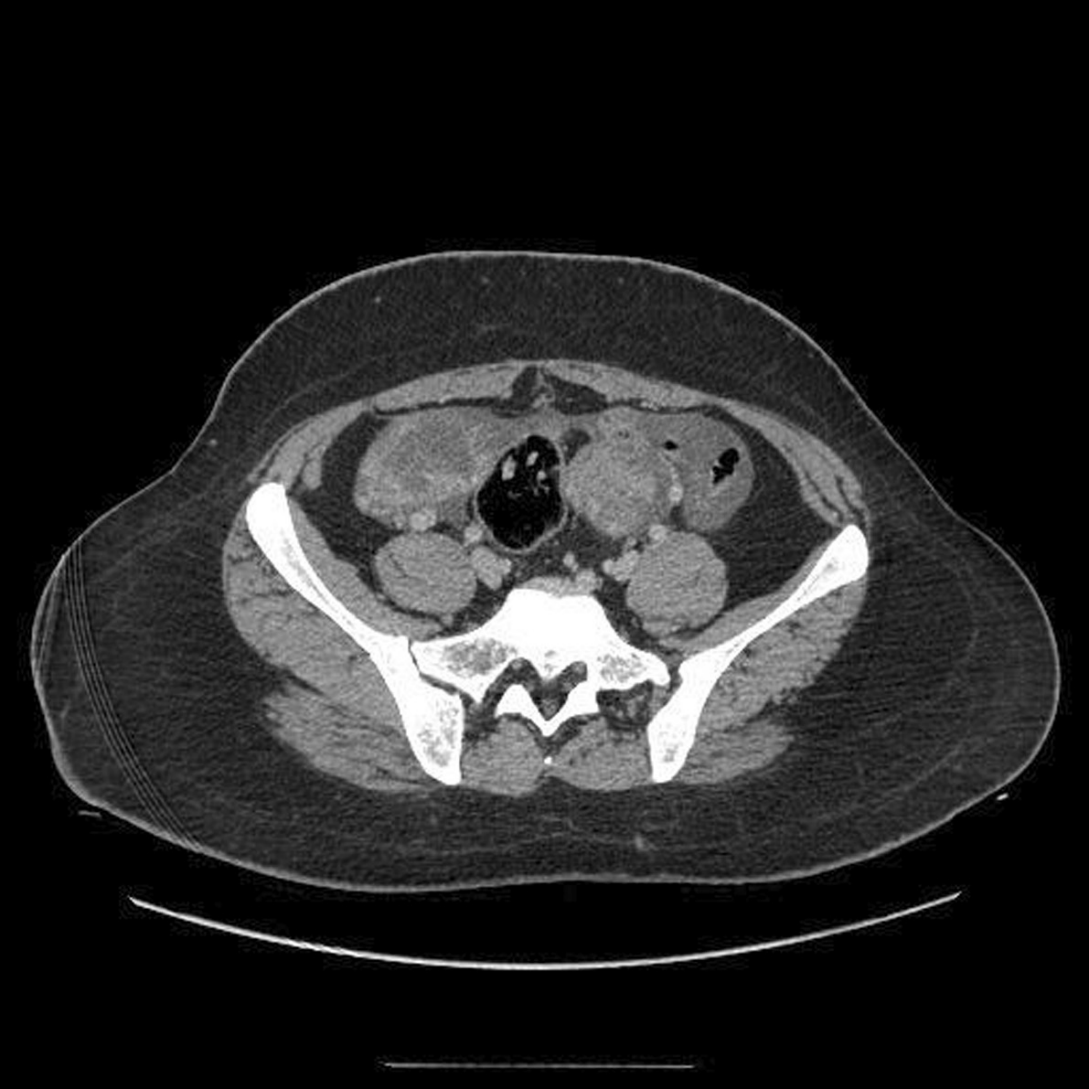
Pelvic CT scan showcasing bilateral ovarian mass

**FIGURE 2:**
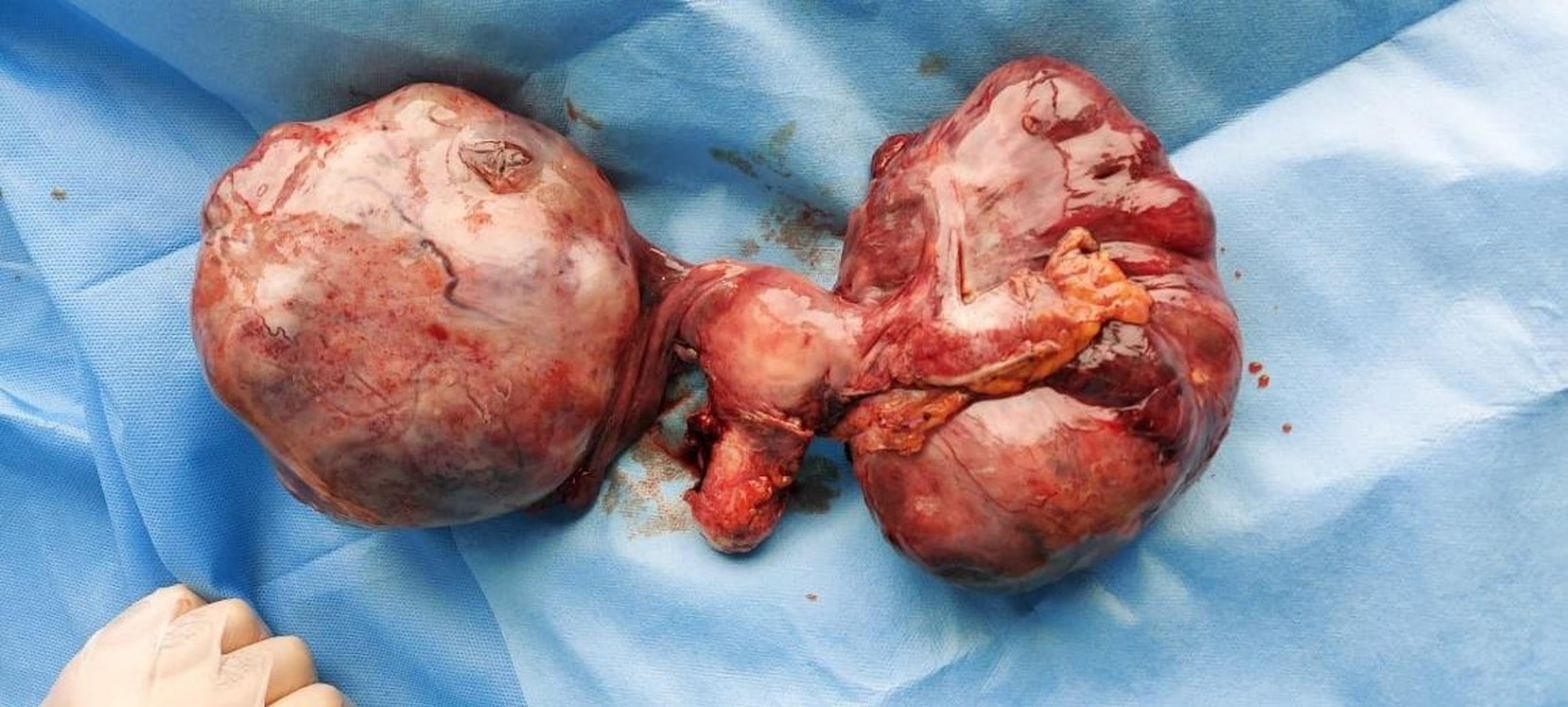
Bilateral ovarian masses and uterus after TAH‐BSO

**FIGURE 3:**
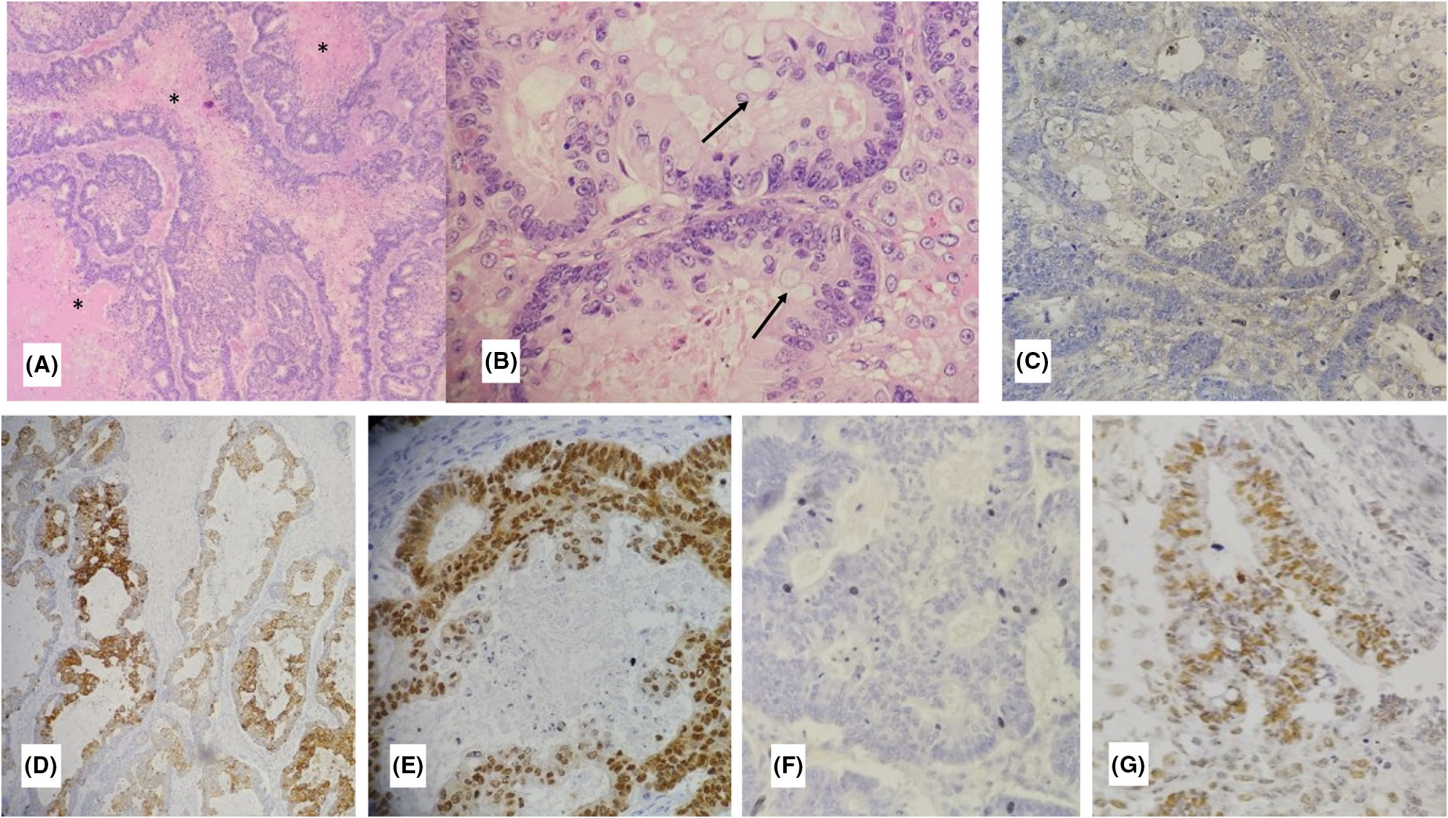
Microscopic examination of hematoxylin and eosin slides show (A) large and irregular glandular structures with intraluminal dirty necrosis (stars) (100×), (B) stratified columnar epithelial lining with atypical vesicular nuclei and occasional goblet cells (arrows) (400×). IHC study shows (C) negative result for CK7, (D and E) positive reaction with CK20, and CDX2, (F) negative result with PAX8, ER, PR and (G) intact nuclear expression of MLH1, PMS2, MSH2, and MSH6

The case was a 26‐year‐old nulliparous woman who was first presented with bilateral abdominal mass palpation and distention. She stated that she had experienced no weight loss or rectal or vaginal bleeding. Her menstrual periods were also regular without any menorrhagia. Upon physical examination, bilateral mass was palpated, but no there were no signs of guarding, tenderness, or rebound tenderness. Hence, the patient underwent ultrasonographic imaging, which showed two huge solid cystic masses in the left and right adnexa. A CT scan was also obtained, which showed cystic masses (Rt: 136*116, Lt: 116*108) in the right and left ovary with no seedings or ascites (Figures 1 and 2). This unusual presentation was unusual in primary ovarian tumors; therefore, a metastasis origin was suspected. The patient underwent a needle biopsy of the right ovary, and an immunohistochemistry evaluation revealed extra‐ovarian source (CK20 and CDX2 expression; Figure 3). After that, the patient underwent colonoscopy and upper endoscopy that showed a circumferential ulcerated mass in the sigmoid, according to the pathology results; the patient underwent total colectomy, total abdominal hysterectomy, and bilateral salpingo‐oophorectomy. During the surgery, after the masses were removed, the entire abdominal cavity was explored for seeding and other sites were inconclusive metastatic. The patient showed cancer involvement in two organs, so we also ruled out lynch syndrome with microsatellite instability testing and IHC results.

## 3 DISCUSSION

Our case was a young woman who was first presented with asymptomatic bilateral ovarian masses until they grew in size and caused distention.

Metastatic ovarian cancers are not very common, and 4.7% of ovarian cancers are metastatic.^1^, ^7^ Accordingly, it has been estimated that over half of them stem from gastrointestinal tract.^3^ It is of utmost importance to distinguish the origin of cancer as the evaluative workup and treatment plans can substantially differ. Since the morphologic features and IHC profiles of each distinct type of carcinoma are non‐specific and overlapping, other features such as surface involvement, presence of signet ring, and nodular involvement are used to distinguish these pathologies.^3^, ^5^, ^8^, ^9^

Colorectal carcinomas are the third most prevalent cancer in the United States, and while many cases are asymptomatic, screening tests such as a workup for iron deficiency anemia or rectal bleeding could help detect these cancers at early stages; meanwhile, these cancers can also cause abdominal pain or intestinal obstruction or perforation.^3^, ^9^, ^10^ Our case stated that she only felt a palpable mass in both ovaries, and she had no gastrointestinal‐related symptoms. Colorectal carcinomas are classified into two categories of non‐polypoid and intramucosal polypoid growth. The overall prevalence of invasion in colorectal carcinoma is lower than in gastric carcinomas, and it is more prevalent in non‐polypoid types as the gross morphology is suggestive of deeper infiltration of tumors.^3^, ^8^, ^9^, ^10^ Interestingly, our case showed solitary metastasis of adenocarcinoma in ovaries; no other organ was involved. This rare presentation and the lack of GI‐related symptoms led to the fact that cancer could only be identified using histopathology and IHC. The current consensus states that preoperative staging can significantly help in planning. Cases such as this indicate that a thorough investigation using MRI and CT scan could help in staging the disease; furthermore, certain tumor markers such as CA19‐9, CEA, and particularly CA242 could provide a more specific diagnosis and staging.^3^, ^7^, ^8^, ^11^, ^12^ The current consensus in regard to surgical intervention indicates Hemi to total colectomy in respect to the level of involvement.^8^, ^10^, ^13^, ^14^, ^15^

Concurrent colorectal carcinoma and ovarian cancer suggest Lynch syndrome, which can affect other organs such as the urinary tract, skin, and brain. This syndrome is diagnosed using molecular genetic identification of pathogenic MLH1, MSH2, MSH6, PMS2 genes, or EPCAM deletion. The workup, in this case, ruled this cancer out by microsatellite instability testing and IHC results.^6^, ^13^, ^14^

Morphologic and histopathologic features of the tumor in this case and bilateral ovarian involvement are characteristic of extra‐ovarian metastatic carcinoma, especially colorectal origin. However, primary ovarian mucinous and endometrioid carcinomas may exhibit similar morphologic findings. IHC study results showed positive reaction with CK20 and CDX2 and negative staining with CK7, PAX8, ER, and PR confirmed the colorectal origin of metastatic carcinoma. Although the presence of dirty necrosis decreases the possibility of MSI‐H,^16^ due to the young age of the patient, IHC study for mismatch repair proteins was done. All of them show a regular pattern of expression, representing the low probability of MSI‐H or lynch syndrome. Extra GI tumor could represent metastatic colorectal cancer, and it is essential to identify the origin of carcinomas. Meanwhile, lynch syndrome, which presents itself with similar symptoms, needs to be ruled out using IHC and MSI. It is also essential to explore the abdominal cavity meticulously to rule out any form of seeding.

## AUTHOR CONTRIBUTION

MA and AH contributed to developing the research idea and composing and revising the manuscript. BB contributed to composing and revising the manuscript. FN contributed to developing the research idea and revising the manuscript.

## FUNDING INFORMATION

None.

## CONFLICT OF INTEREST

The authors have no conflict of interest to declare.

## ETHICAL APPROVAL

This study was approved by the research and ethics committee of the Tehran University of Medical Sciences.

## CONSENT

Written informed consent was obtained from the patient's next of kin to publish this case report and any accompanying images. The patient's family has given their informed consent to publish this case.

## Data Availability

Data sharing is not applicable to this article as no datasets were generated or analyzed during the current study.
